# Incorporating ex-vivo lung perfusion into the UK adult lung transplant service: an economic evaluation and decision analytic model

**DOI:** 10.1186/s12913-019-4154-6

**Published:** 2019-05-22

**Authors:** N. McMeekin, A. E. Chrysos, L. Vale, A. J. Fisher

**Affiliations:** 1 HEHTA, Institute of Health and Wellbeing, University of Glasgow, Glasgow, UK; 2 Health Economics Group, Institute of Health & Society, Newcastle University, Newcastle upon Tyne, UK; 3 Population Health Sciences, Bristol Medical School, Canynge Hall, University of Bristol, Bristol, UK; 4 Institute of Transplantation, Newcastle upon Tyne Hospitals NHS Foundation Trust, Newcastle upon Tyne, UK; 5 Institute of Cellular Medicine, Newcastle University, Newcastle upon Tyne, UK

**Keywords:** Ex-vivo lung perfusion, EVLP, Lung transplantation, Lung transplant waiting list, Cost-effectiveness, Cost utility analysis, Markov model, Decision analytic model, Economic evaluation

## Abstract

**Background:**

An estimated 20–30% of end-stage lung disease patients awaiting lung transplant die whilst on the waiting list due to a shortage of suitable donor lungs. Ex-Vivo Lung Perfusion is a technique that reconditions donor lungs initially not deemed usable in order to make them suitable for transplantation, thereby increasing the donor pool. In this study, an economic evaluation was conducted as part of DEVELOP-UK, a multi-centre study assessing the clinical and cost-effectiveness of the Ex-Vivo Lung Perfusion technique in the United Kingdom.

**Methods:**

We estimated the cost-effectiveness of a UK adult lung transplant service combining both standard and Ex-Vivo Lung Perfusion transplants compared to a service including only standard lung transplants. A Markov model was developed and populated with a combination of DEVELOP-UK, published and clinical routine data, and extrapolated to a lifetime horizon. Probabilistic sensitivity and scenario analyses were used to explore uncertainty in the final outcomes.

**Results:**

Base-case model results estimated life years gained of 0.040, quality-adjusted life-years (QALYs) gained of 0.045 and an incremental cost per QALY of £90,000 for Ex-Vivo Lung Perfusion. Scenario analyses carried out suggest that an improved rate of converting unusable donor lungs using Ex-Vivo Lung Perfusion, similar resource use post-transplant for both standard and EVLP lung transplant and applying increased waiting list costs would reduce ICERs to approximately £30,000 or below.

**Conclusion:**

DEVELOP-UK base-case results suggest that incorporating Ex-Vivo Lung Perfusion into the UK adult lung transplant service is more effective, increasing the number of donor lungs available for transplant, but would not currently be considered cost-effective in the UK using the present NICE threshold. However, results were sensitive to change in some model parameters and in several plausible scenario analyses results indicate that a service incorporating Ex-vivo lung perfusion would be considered cost-effective .

**Trial registration:**

ISRCTN registry number: ISRCTN44922411.

Date of registration: 06/02/2012.

Retrospectively registered.

## Background

Respiratory disease accounts for one in five deaths in the UK [1]. Lung transplantation provides the only life prolonging treatment option for a select few patients with end-stage lung disease. Whilst not all those with end stage lung disease might be suitable for lung transplantation, a shortage of suitable donor lungs results in around 20–30% of patients dying whilst on the lung transplant (LTx) waiting list [2]. Although the number of potential donors is limited, the primary cause of this shortage is that approximately 80% of potential donor lungs are felt to be unusable for standard LTx due to irreversible pre-existing lung disease or by reversible damage occurring during end-of-life care [3].

One solution to this problem is Ex-Vivo Lung Perfusion (EVLP), a novel technique used to increase the existing donor pool by assessing and reconditioning donor lungs felt to be unusable in order to make them clinically safe for LTx [4]. These donor lungs are attached to the EVLP apparatus, ventilated, warmed to body temperature and flushed with perfusate, donor lungs are then assessed for suitability for transplant. The first successful EVLP LTx took place in 2005 [5] and by 2014, around 350 EVLP transplants had taken place worldwide [6]. Initial experience has shown that EVLP can increase the number of LTxs by 15 to 30% [6]. However, a definitive study would be needed to establish the safety, effectiveness and cost-effectiveness of this technique compared with standard transplant.

### Summary of DEVELOP-UK

DEVELOP-UK was a multi-centre, unblinded, non-randomized, non-inferiority observational study that sought to evaluate the clinical- and cost-effectiveness of the EVLP technique compared with standard lung transplantation in the UK [4]. Participants were adults (≥18 years) who had already been accepted onto the UK LTx waiting list. The target sample size was 306 in the standard transplant arm and 102 in the EVLP arm. Non-inferiority was assumed if the hazard rate of death during the first year was not more than doubled by the use of EVLP.

Four hundred and eighty-seven participants were recruited into the study, 202 received a LTx; 184 standard and 18 EVLP. The small numbers in the EVLP arm led to the study being terminated before the recruitment targets were reached. Baseline characteristics of transplant recipients in each arm are presented in Table 1. Results of the study showed that a third of the donor lungs subjected to EVLP were deemed suitable for transplant (18 out of 53) representing a 10% increase in existing standard LTx activity due to EVLP transplants. One-year survival in the EVLP arm was lower than in the standard arm, 67% compared to 80%. However, the non-inferiority definition of the study was satisfied. The small numbers of individuals receiving transplants means that the study was underpowered and non-inferiority was not established. An unexpected consequence during the study was an increase in standard LTxs; one centre witnessed an increase of 25%. Overall in the UK, the increase in the number of LTxs was 6.9% in the first year of the trial (2012/13), and the increase from pre-trial was 20% in the second year (2013/14). In the year after the trial finished (2014/15), the number of transplants returned to almost pre-trial rates; an increase of only 1.7% compared to the levels pre-trial [7]. The proposed economic evaluation was a simple head-to-head comparison of EVLP compared to standard lung transplantation, not accounting for any spill-over effects. The cost of an EVLP transplant was approximately £35,000 higher than a standard LTx, and quality-adjusted life-years (QALYs) were similar in both arms twelve months after transplantation [4].

**Table 1:** Recipient baseline characteristics

Recipient Characteristic		EVLP *N* = 18	STANDARD *N* = 184	Total *N* = 202
Gender	Male n (%)	13 (72.2)	106 (57.6)	119 (58.9)
	Female n (%)	5 (27.8)	78 (42.4)	83 (41.1)
Age (Years)	n	18	183	201
	Missing	0	1	1
	Median	56	51	52
	IQR	46–59	38–58	38–58
	Range	20–64	18–70	18–70
Diagnosis	COPD n (%)	5 (27.8)	40 (21.7)	45 (22.3)
	Cystic Fibrosis n (%)	4 (22.2)	47 (25.5)	51 (25.2)
	Interstitial Lung Disease n (%)	7 (38.9)	47 (25.5)	54 (26.7)
	Emphysema n (%)	0 (0)	26 (14.1)	26 (12.9)
	Non-CF Bronchiectasis n (%)	1 (5.6)	8 (4.3)	9 (4.5)
	Obliterative Bronchiolitis n (%)	0 (0)	2 (1.1)	2 (1.0)
	Pulmonary Hypertension n (%)	1 (5.6)	3 (1.6)	4 (2.0)
	Other n (%)	0 (0)	9 (4.9)	9 (4.5)
	Missing n (%)	0 (0)	2 (1.1)	2 (1.0)
Diabetes	Yes n (%)	4 (22.2)	33 (18.1)	37 (18.3)
	No n (%)	13 (72.2)	142 (78.0)	155 (76.7)
	Missing n (%)	1 (5.6)	9 (3.9)	10 (5.0)
BMI	n	17	182	199
	Missing	1	2	3
	Median	21.6	23.8	23.7
	IQR	18.4–26.3	20.5–26.5	20.4–26.5
	Range	17.6–32.5	15.4–34.2	15.4–34.2
FEV1 (L)	n	15	176	191
	Missing	3	8	11
	Median	1.2	0.9	0.9
	IQR	0.7–1.9	0.6–1.4	0.6–1.5
	Range	0.5–2.5	0.3–3.6	0.3–3.6
FEV1%	n	15	171	186
	Missing	3	13	16
	Median	29	26	27
	IQR	22–50	20–45	20–45
	Range	15–67	11–105	11–105
Type of Transplant	Single n (%)	2 (11.1)*	24 (13.0)**	26 (13)
	Bilateral n (%)	16 (88.9)	152 (82.6)	168 (83)
	Missing n (%)	0 (0) * 1 left, 1 right	8 (4.4) **9 left, 13 right, 2 NK	8 (4)
Cardiopulmonary Bypass Use	No n (%)	2 (11.1)	46 (25.0)	48 (24)
	Yes n (%)	16 (88.9)	116 (63.0)	132 (65)
	Not known n (%)	0 (0)	22 (12.0)	22 (11)
*Source*[4]				

*BMI* body mass index, *CF* cystic fibrosis, *COPD* chronic obstructive pulmonary disease, *EVLP* Ex-vivo lung perfusion, *FEV* forced expiratory volume, *IQR* inter quartile range, *NK* not known

The main outcome of DEVELOP-UK was survival at 12 months post-transplant. This was 0.67 (95% Confidence Interval (CI) 0.4–0.83) in the EVLP arm and 0.80 (95% CI 0.74–0.85) in the standard arm. Given the modest sample sizes a formal comparison was not attempted.

The mean cost of each EVLP procedure (reconditioning of donor lungs only) was approximately £14,500 per patient. However, EVLP patients experienced increased length of stay in the intensive care unit and needed more lung support post-transplant resulting in increased post-operative costs of £22,000 compared to standard transplant. Overall the cost of an EVLP LTx was on average around £35,000 greater than a standard LTx (using a 1:1 conversion rate).

More detail on DEVELOP-UK results can be found in the full study report [4].

### Aims

The aim of this economic evaluation was to evaluate the cost-utility of EVLP transplants within the current UK National Health Service (NHS) LTx service, and to assess whether including EVLP can ease waiting lists. A model of the adult LTx service providing both standard and EVLP transplants was compared with a service including only standard transplants. The decision-analytic model was populated with DEVELOP-UK, published and clinical routine data.

## Methods

### Analysis overview

The target population was adults on the UK LTx waiting list, while the perspective was the UK NHS. Following National Institute for Health and Care Excellence (NICE) guidelines, the time horizon used was lifetime to capture all costs and outcomes over a patient’s lifetime, with a 3.5% discount rate for both costs and health benefits [8]. Cost-effectiveness was measured against the current NICE threshold of £20,000 to £30,000 per QALY [9].

Interventions were a UK adult LTx service combining both EVLP and standard LTxs (‘EVLP service’) compared to a standard LTx only service (‘Standard service’). These interventions were chosen to illustrate the effect of introducing EVLP into the existing UK LTx service as a means of increasing the donor lung pool.

### Model structure

**Fig. 1:**
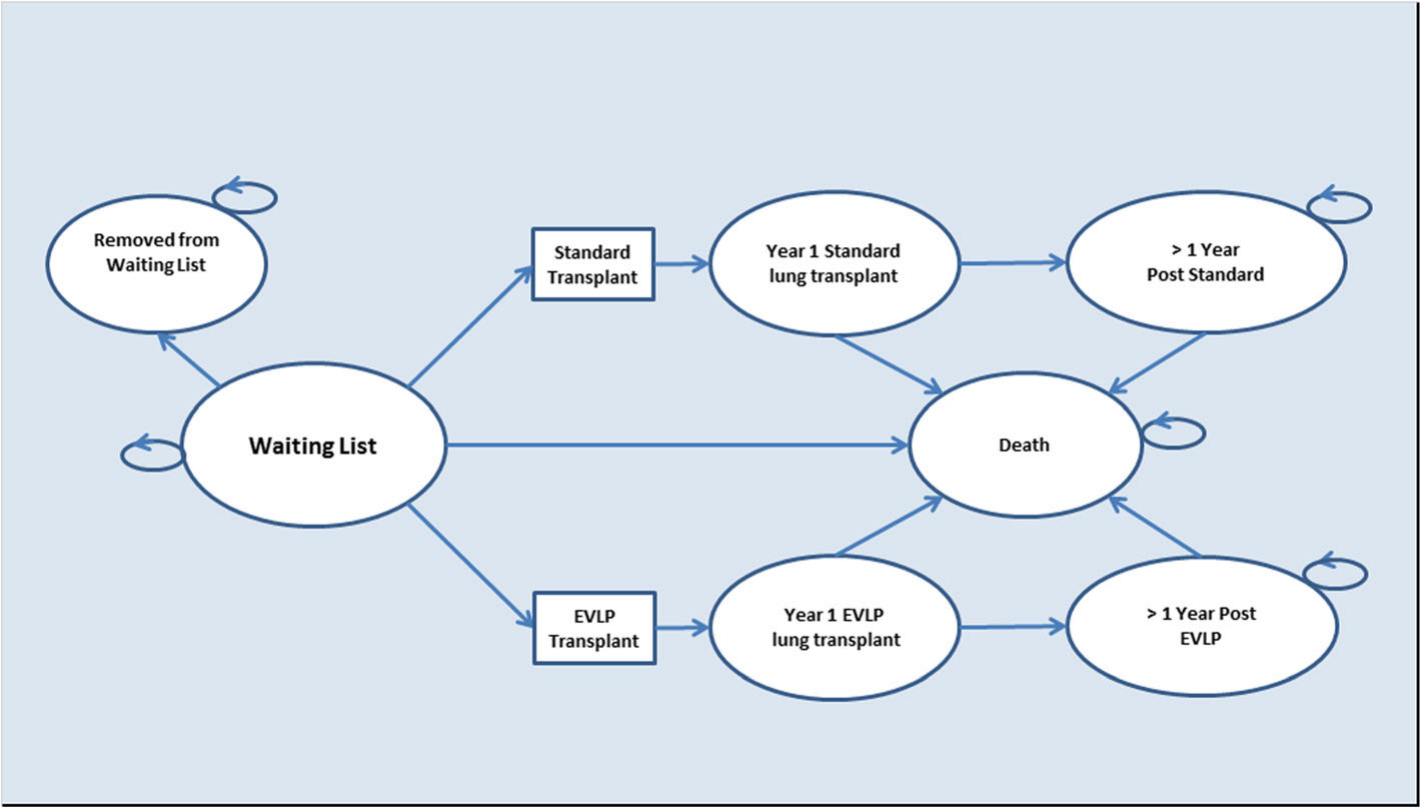
Model structure

A Markov model (Fig. 1) was developed using Microsoft® Excel® 2010 (Microsoft, Washington, USA) to extrapolate DEVELOP-UK results to a lifetime horizon. The model represents the flow through the UK adult LTx service; beginning in the ‘Waiting list’ state, the patient may remain or progress to one of the following states; ‘Removed from the waiting list’, ‘Death’; ‘Receiving a standard LTx’; or ‘Receiving an EVLP LTx’. Post-transplant, the patient progresses either to death or to the relevant post one-year transplant state. Cycle length was 1 year reflecting the clinically important first year following survival [10]. Costs and outcomes were assigned to each model state.

### Model transitions

Transitions from the waiting list were taken from figures published by NHS Blood and Transplant (NHSBT) [11]. A cohort of 251 patients who registered for an LTx between 1 April 2011 and 31 March 2012, were followed for 3 years, covering the same time period of DEVELOP-UK trial. The numbers of patients who left the waiting list each year were used to calculate transition rates.

Survival figures derived from DEVELOP-UK were not deemed robust enough for use as a model parameter due to the small number of participants in the EVLP arm and the short period of follow-up (12 months). Therefore, survival post-transplant was determined using NHSBT survival data reported for a cohort of 348 patients who received their LTx between 1 April 2004 and 31 March 2006 [12] There is no evidence in the literature, nor from DEVELOP-UK, of any difference in survival post-transplant between standard and EVLP transplants [6], so the same survival was assumed for both types of transplant. DEVELOP-UK data showed a non-significant trend towards higher mortality for EVLP, however, it is important to note that due to the small sample sizes in DEVELOP-UK the data are not sufficiently comprehensive to permit a robust comparison.

One-year survival was used for ‘Year 1’ following both standard and EVLP transplants. For post ‘Year 1’ states an estimated mean post-transplant survival, which was determined using area-under-the-curve methods precluding the need for tunnel states, was applied to each transplant recipient. This was calculated using survival data at 3, 5 and 10 years post-transplant and assuming 25 years to be maximum life expectancy post-transplant (data from the International Society for Heart and Lung Transplantation (ISHLT) report that for adults receiving an LTx between January 1990 and June 2012 survival of 19 years post-transplant is 12% [13]).

### Model assumptions

- The ‘Removed from waiting list’ state is an absorbing state, where a patient does not accrue costs or utilities (the probability attached to transitioning into this state is independent of transplant type). Patients transition to this state when they are too ill to receive a transplant, prognosis is extremely poor for these patients, with survival typically less than a month, Exploratory analysis was carried out to estimate the effects of this assumption, by including costs and QALYs for patients in this state and varying the survival between 1, 3 and 6 months.
- NHSBT waiting list transitions were only available for three years post-registration. As only 5% of the cohort remained on the waiting list beyond three years, it was assumed that transitions remain constant from year three onwards.
- It was assumed that utilities at 12 months post-transplant, derived from DEVELOP-UK data, were the same for 2 years post-transplant and onwards. This assumption is backed up by previous research where utilities post double-lung transplant were similar for 7–18 months (0.83), 19–36 months (0.81) and > 36 months (0.82) [14].

### Outcomes

The principle outcome measure used was QALYs, a measure combining quality and length of life, calculated using the patients’ responses to the 36-item Short Form Health Survey (SF-36) [15]. DEVELOP-UK participants completed SF-36 questionnaires whilst on the waiting list and at 3 and 12 months post-transplant. SF-36 responses were converted into SF-6D scores using the SF-6D algorithm; Values of 0 represent death, and 1 represents perfect health. Utility scores post-transplant were combined with length of survival to calculate the estimated QALYs for each patient [16]. Data were combined for the EVLP and standard transplants giving health-state specific utilities, not varying by type of transplant.

### Costs

Costs were calculated based on resource use during DEVELOP-UK [4]. Case report forms were used along with expert opinion to identify resource use within eight stages of lung transplantation; donor hospital, lung retrieval, transplant preparation, EVLP procedure, transplant surgery, post-operative care, outpatient care, and concomitant medications. Unit costs were obtained from the British National Formulary(BNF) [17], Personal Social Services Research Unit(PSSRU) [18], NHS reference costs [19], Information Services Division Scotland(ISD) [20], direct company quotes, expert opinion, hospital costing tools and medical suppliers websites. The price year used was 2016/2017 (and the currency was pounds sterling (GBP, £).

Total mean cost per participant was calculated by summing the products from unit costs multiplied by the quantities of resources used, then dividing by the number of participants per arm.

To calculate the cost for the year of transplant all costs listed above were included. For the years following, transplant outpatient and medication costs were summed and decreased annually in line with costs published by Anyanwu and colleagues [14].

During DEVELOP-UK, 53 EVLP assessments were completed resulting in 18 EVLP transplants, a conversion rate of 53:18. To reflect this in the micro-costing analysis, EVLP transplant costs were calculated by applying a 53:18 ratio to costs of the first four stages outlined above as these stages are different between the two LTx procedures. However, this ratio was not available for the standard arm and therefore, this was considered to be equal to 1:1.

Waiting list costs were not available using DEVELOP-UK data and consequently they were obtained from a published economic evaluation of adult lung transplantation in the UK [14]. Costs for double lung transplantation waiting list were used as 83% of transplants in DEVELOP-UK were double-lung, and inflated to 2016/2017 price year using the Hospital and Community Health Services Index [21, 22].

Details of the costs used in the model can be found in the DEVELOP-UK report [4].

### Assessment of cost-effectiveness

An incremental cost-effectiveness ratio (ICER) was calculated by dividing the difference in total mean costs by the difference in their mean outcomes between the two arms.

### Uncertainty

Lack of robustness in DEVELOP-UK data as well as model assumptions and parameters created uncertainty. Because of this uncertainty, the use of point estimates to populate the model would be potentially misleading. For this reason, probabilistic sensitivity analysis (PSA) was used to estimate mean costs and QALYs for the analysis. Suitable distributions were assigned to each parameter and de facto standard 1000 simulations were carried out. Best practice was followed for PSA and the choice of parameters [23]. Model parameters and distributions are listed in Table 2, and an incremental cost-effectiveness plane and a cost-effectiveness acceptability curve (CEAC) are presented. In Table 2 the key difference in transition probabilities between EVLP and standard lung transplant is that for any given time period there is a 10% increase in the number of transplants performed. The impact of this in the model is that fewer patients will die whilst on the waiting list or transfer off the waiting list (into end of life care).

**Table 2:** Base case model parameters

Parameters	Mean	Alpha/Beta	Distribution	Source
Model transitions				
From ‘Waiting list’ to ‘Removed from waiting list’ (Standard and EVLP service)				
*Year 1*	0.020	5.02, 245.98	Beta	NHSBT [11]
*Year 2*	0.030	2.51, 80.32	Beta	NHSBT [11]
*Year 3 onwards*	0.077	2.51, 30.12	Beta	NHSBT [11]
From ‘Waiting list’ to Transition to ‘Death’ (Standard and EVLP service)				
*Year 1*	0.150	37.65, 213.35	Beta	NHSBT [11]
*Year 2*	0.212	17.57, 65.26	Beta	NHSBT [11]
*Year 3 onwards*	0.077	2.51, 30.12	Beta	NHSBT [11]
From ‘Waiting list’ to ‘Receiving a standard lung transplant’				
*Year 1*	0.510	128, 122.99	Beta	NHSBT [11]
*Year 2*	0.364	30.1, 52.71	Beta	NHSBT [11]
*Year 3 onwards*	0.462	15.1, 17.57	Beta	NHSBT [11]
Increase in lung transplant activity due to EVLP procedure (Applied in addition to standard LTx transition in EVLP service arm)	10%	18, 184	Beta	DEVELOP-UK data [4]
Post lung transplant survival				
One-year survival for standard and EVLP	0.77	257.91, 77.04	Beta	UK cardio audit [27]
Area under the curve				
*1-year survival*	0.77	257.91, 77.04	Beta	UK cardio audit [27]
*3-year survival*	0.7	225.19, 96.51,	Beta	UK cardio audit [27]
*5-year survival*	0.53	167.15, 148.23	Beta	UK cardio audit [27]
*10-year survival*	0.34	116.90, 226.93	Beta	UK cardio audit [27]
Utilities				
Waiting list	0.563	2292.2, 1782.4	Beta	DEVELOP-UK data [4]
1-year post standard lung transplant	0.702	465.8, 197.3	Beta	DEVELOP-UK data [4]
2 years onwards post standard lung transplant	0.734	439.7, 159.4	Beta	DEVELOP-UK data [4]
1-year post EVLP lung transplant	0.702	465.8, 197.3	Beta	DEVELOP-UK data [4]
2 years onwards post EVLP transplant	0.734	439.7, 159.4	Beta	DEVELOP-UK data [4]
Costs				
Waiting list cost per year	£23,829	103.1, 239.3	Gamma	Anyanwu [14]
Standard lung transplant	£51,778	304.2, 175.5	Gamma	DEVELOP-UK data [4]
EVLP lung transplant	£137,527	98.4, 1441.1	Gamma	DEVELOP-UK data [4]
Post lung transplant costs – standard LTx (outpatient and concomitant medication Table 38 HTA report[4])				
Year 1	£9700	93.5, 107.1	Gamma	DEVELOP-UK data [4]
Year 2	£3812	93.4, 42.1	Gamma	DEVELOP-UK data [4]
Year 3 onwards	£3506	93.4, 38.7	Gamma	DEVELOP-UK data [4]
Post lung transplant costs – EVLP LTx				
Year 1	£5919	20.2, 302.7	Gamma	DEVELOP-UK data [4]
Year 2	£2326	20.2, 118.9	Gamma	DEVELOP-UK data [4]
Year 3 onwards	£2140	20.2, 109.4	Gamma	DEVELOP-UK data [4]

N.B. All parameters used are for a double lung transplantation where data was available for both single and double lung transplant

*EVLP Ex-vivo lung perfusion, *LTx* Lung transplantation, *NHSBT* NHS blood and transplant

Scenario analysis investigated uncertainty by using plausible alternative parameters and analysing the effect on costs and utilities. Nine scenario analyses were carried out. The first used NHSBT transition rates from before the trial. These rates were from a cohort of patients added to the waiting list between 1 April 2008 and 31 March 2009 [24]. Using these transitions excludes any effects that the change in retrieval procedure (for example, unexpected increase in standard LTx) during DEVELOP-UK may have had on transition rates. The second scenario evaluated effects of raising transplant activity due to EVLP to 20%, a conservative estimate [6]. The third and fourth scenarios altered the DEVELOP-UK EVLP conversion rate (base-case: 53:18). A conversion rate of 45% was chosen in the third scenario because prior to DEVELOP-UK the conversion rate was anticipated to be 40–50%, while the fourth scenario used a 1:1 rate to mirror the standard lung transplant conversion rate used. No relevant published economic evaluations of EVLP were found so there was no prior work regarding retrieval to transplant conversion rates in this area. The fifth scenario used published utilities from UK LTx patients [25]. These utilities were for a larger cohort than DEVELOP-UK; 87 waiting list patients; 255 transplant recipients, and although only standard LTx patient utilities were included, these were used for EVLP patients too. The sixth scenario excluded EVLP transplants completely focussing instead on an unexpected increase in standard LTx activity witnessed during DEVELOP-UK. The seventh scenario replaced UK Cardiothoracic Transplant Audit figures for 12-month survival post-transplant with 12-month survival from DEVELOP-UK. The eighth scenario used the same post-transplant inpatient care costs in both arms to explore excluding the increase in post-transplant inpatient costs seen in DEVELOP-UK, but not reported in other EVLP settings [26]. The last scenario increased the waiting list costs by 120%; waiting list costs were taken from a UK study published in 2002, uplifted to 2016/17, waiting list costs are likely more expensive now than when the 2002 study was carried out due to the availability of high costs therapeutics for cystic fibrosis (CF) and idiopathic pulmonary fibrosis (IPF). Model parameters and distributions used in all scenario analyses are presented in Table 3.

**Table 3:** Scenario analysis parameters

Parameters	Mean	Alpha/Beta	Distribution	Source
Scenario 1: Pre-trial model transitions				
From ‘Waiting list’ to ‘Removed from waiting list’ (Standard and EVLP service)				
*Year 1*	0.08	16, 180	Beta	NHSBT [24]
*Year 2*	0.03	2, 67	Beta	NHSBT [24]
*Year 3 onwards*	0.11	3, 32	Beta	NHSBT [24]
From ‘Waiting list’ to Transition to ‘Death’ (Standard and EVLP service)				
*Year 1*	0.17	33,163	Beta	NHSBT [24]
*Year 2*	0.09	6, 63	Beta	NHSBT [24]
*Year 3 onwards*	0.17	6, 29	Beta	NHSBT [24]
From ‘Waiting list’ to ‘Receiving a standard lung transplant’				
*Year 1*	0.4	78, 118	Beta	NHSBT [24]
*Year 2*	0.38	26, 43	Beta	NHSBT [24]
*Year 3 onwards*	0.28	10, 25	Beta	NHSBT [24]
Scenario 2: 20% increase in lung transplant activity due to EVLP procedure				
Increase in LTx activity due to EVLP procedure (Applied in addition to standard LTx transition in ‘EVLP service’ arm)	0.2	36, 184	Beta	DEVELOP-UK data [4]
Scenario 3: 45% Conversion rate				
EVLP lung transplant	£119,672	75.1, 1644.4	Gamma	DEVELOP-UK data [4]
Scenario 4: 1:1 conversion rate				
EVLP lung transplant	£89,455	42.4, 2174.3	Gamma	DEVELOP-UK data [4]
Scenario 5: Utilities from literature				
Waiting list	0.31	59.72, 132.93	Beta	Anyanwu et al. [25]
1-year post standard lung transplant	0.83	1099.12, 257.82	Beta	Anyanwu et al. [25]
2 years onwards post standard lung transplant	0.82	854.12, 187.49	Beta	Anyanwu et al. [25]
1-year post EVLP lung transplant	0.83	1099.12, 257.82	Beta	Anyanwu et al. [25]
2 years onwards post EVLP transplant	0.82	854.12, 187.49	Beta	Anyanwu et al. [25]
Scenario 6: Increase in LTx activity (Excluding EVLP)				
*Pre-trial*				
From ‘Waiting list’ to ‘Removed from waiting list’				
*Year 1*	0.08	16, 180	Beta	NHSBT [11]
*Year 2*	0.03	2, 67	Beta	NHSBT [11]
*Year 3 onwards*	0.11	3, 32	Beta	NHSBT [11]
From ‘Waiting list’ to Transition to ‘Death’				
*Year 1*	0.17	33,163	Beta	NHSBT [11]
*Year 2*	0.09	6, 63	Beta	NHSBT [11]
*Year 3 onwards*	0.17	6, 29	Beta	NHSBT [11]
From ‘Waiting list’ to ‘Receiving a standard lung transplant’				
*Year 1*	0.40	78, 118	Beta	NHSBT [11]
*Year 2*	0.38	26, 43	Beta	NHSBT [11]
*Year 3 onwards*	0.28	10, 25	Beta	NHSBT [11]
*Within trial*				
From ‘Waiting list’ to ‘Removed from waiting list’				
*Year 1*	0.020	5.02, 245.98	Beta	NHSBT [24]
*Year 2*	0.030	2.51, 80.32	Beta	NHSBT [24]
*Year 3 onwards*	0.077	2.51, 30.12	Beta	NHSBT [24]
From ‘Waiting list’ to Transition to ‘Death’				
*Year 1*	0.150	37.65, 213.35	Beta	NHSBT [24]
*Year 2*	0.212	17.57, 65.26	Beta	NHSBT [24]
*Year 3 onwards*	0.077	2.51, 30.12	Beta	NHSBT [24]
From ‘Waiting list’ to ‘Receiving a standard lung transplant’				
*Year 1*	0.510	128, 122.99	Beta	NHSBT [24]
*Year 2*	0.364	30.1, 52.71	Beta	NHSBT [24]
*Year 3 onwards*	0.462	15.1, 17.57	Beta	NHSBT [24]
Scenario 7: DEVELOP-UK one-year survival post-transplant				
Standard service	0.80	161.8, 40.4	Beta	DEVELOP-UK [4]
EVLP service	0.67	11.64, 5.73	Beta	DEVELOP-UK [4]
Scenario 8: Same costs of post-transplant inpatient care in both arms (EVLP service = standard service costs plus costs of EVLP procedure)				
Standard service transplant	£51,778	304.2, 175.5	Gamma	DEVELOP-UK data [4]
EVLP service transplant	£95,750	344.74, 269.29	Gamma	DEVELOP-UK data [4]
Scenario 9: Increase in waiting list costs by 120%				
Increase waiting list cost	£50,830	96.04, 529.26	Gamma	120% increase

*EVLP Ex-vivo lung perfusion, LTx Lung transplantation, *NHSBT* NHS blood and transplant

#### Ethics approval and consent to participate to DEVELOP-UK

Ethics approval was received from the National Research Ethics Service (reference number 11/NE/0342) and NHS research and development (R&D) approval was secured prior to commencement of DEVELOP-UK. Local NHS approvals were secured before recruitment commenced at each site. The Newcastle Clinical Trials Unit, in its capacity as study co-ordination centre, obtained a written copy of local approval documentation before initiating each centre and accepting participants into the study.

## Results

### Base-case results

Base-case cost-effectiveness results of the model-based economic evaluation are presented in Table 4. The incremental mean discounted lifetime cost of a LTx was £4010 (95% CI £1863–£6723), higher in the ‘EVLP service’. The incremental life-years gained 0.040 (95% CI -0.011-0.101), higher in the ‘EVLP service’. Incremental QALYs were 0.045 (95% CI 0.007–0.091) higher in the ‘EVLP service’. The incremental cost per life year gained was £100,000 and the incremental cost per QALY gained was £90,000 for the ‘EVLP service’ compared with the ‘Standard service’. 1% of simulations fall below the £20,000 and £30,000 thresholds.

**Table 4:** Base-case cost-effectiveness results

		‘Standard Service’	‘EVLP service’
Discounted	Mean costs	£69,954	£73,964
	Mean life-years gained	5.62	5.66
	Mean QALYs	3.67	3.71
Incremental	Costs		£4010
	Life-years gained		0.040
	QALYs		0.045
ICER	Life-years gained		£100,000
	QALYs		£90,000
Number of	Standard lung transplants	721	675
	EVLP transplants		67

*EVLP Ex-vivo lung perfusion, *ICER* Incremental costs-effectiveness ratio, *QALYs* Quality-adjusted life-years *ICER* incremental cost-effectiveness ratio

In the ‘Standard service’, the number of standard LTxs carried out from a cohort of 1000 was 721. In the ‘EVLP service’ the number of standard LTxs and EVLP LTxs was 675 and 67 respectively, (742 in total). The number of standard LTxs decreased in the ‘EVLP service’ compared with the ‘Standard service’ as a result of the model calculating transitions from the waiting list, as probabilities. In other words, there are fewer patients left in the ‘Waiting list’ state at the end of each cycle in the ‘EVLP service’ due to EVLP transplants, so less patients transition to receive a standard LTx.

**Fig. 2:**
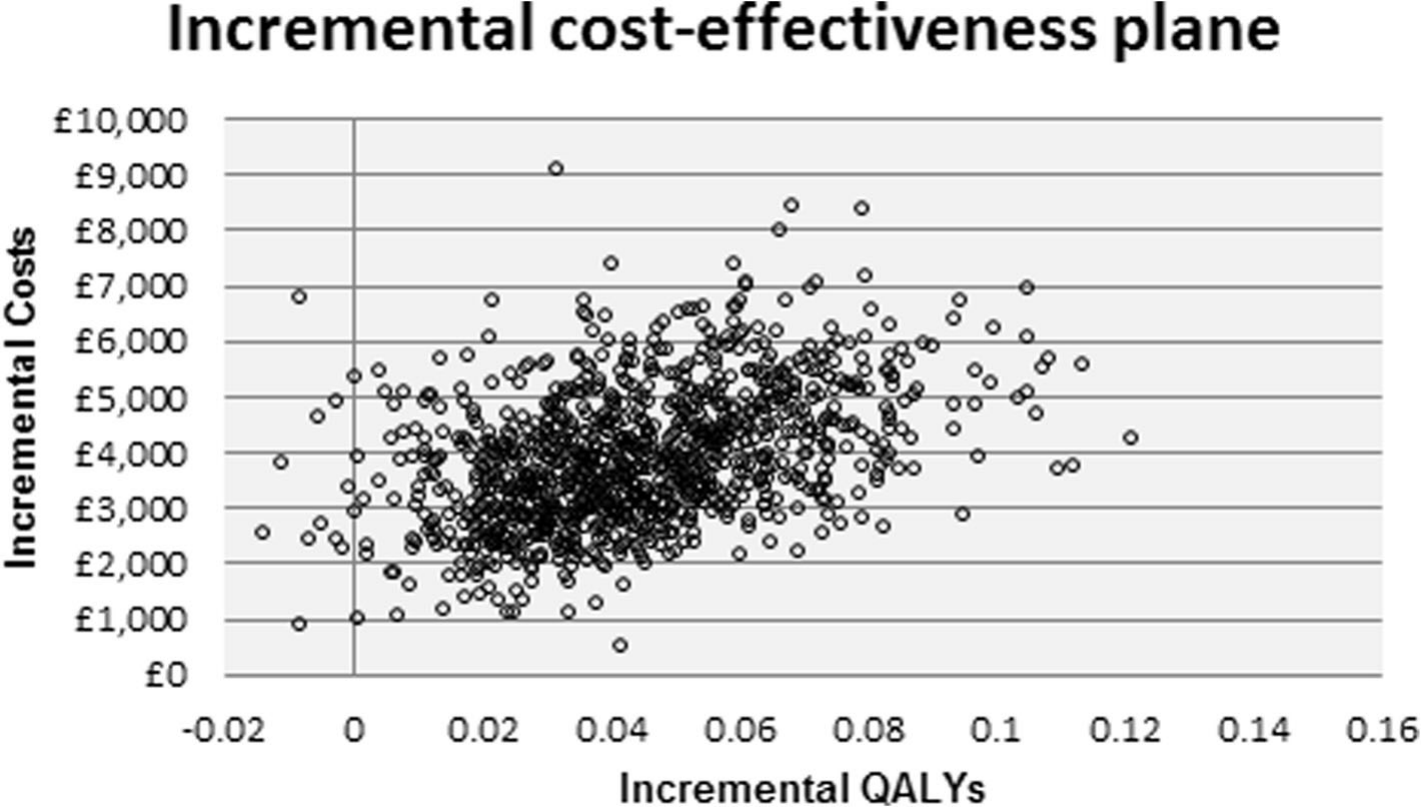
Incremental cost-effectiveness plane: base-case analysis

Incremental costs and QALYs were plotted on an incremental cost-effectiveness plane (Fig. 2). As can be seen, most of the simulations fell in the north-east quadrant where the ‘EVLP service’ had higher costs and more QALYs than the ‘Standard service’. A small number (1.1%) of simulations fell in the north-west quadrant where the ‘EVLP service’ had higher costs and lower QALYs than the ‘Standard service’.

**Fig. 3:**
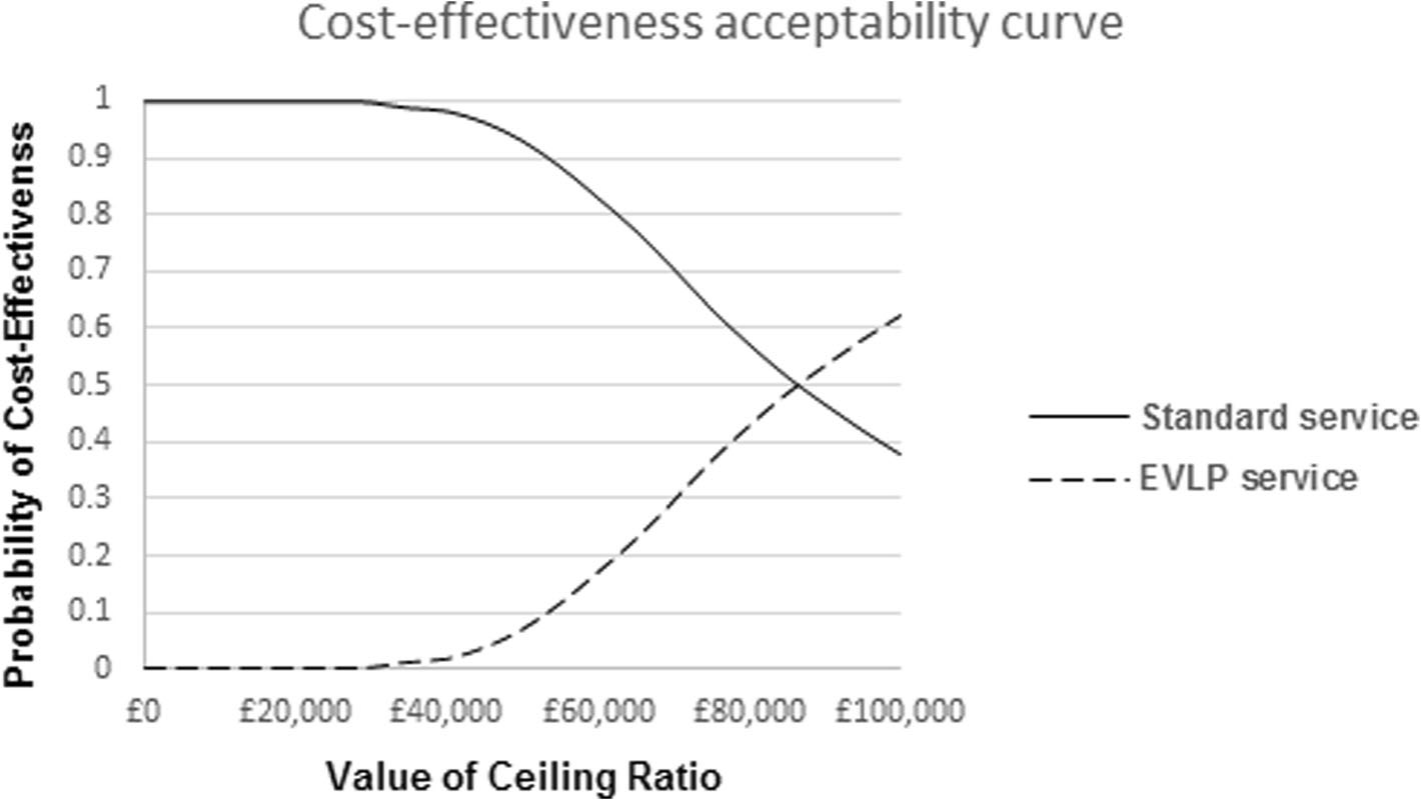
Cost-effectiveness acceptability curve: base-case analysis

The CEAC (Fig. 3) illustrates that there is a 99.9 and 99.8% likelihood of the ‘Standard service’ being more cost-effective than ‘EVLP service’ at a willingness-to-pay of £20,000 and £30,000, respectively. At a willingness-to-pay of £90,000, the ‘EVLP service’ begins to have a higher probability of being more cost-effective than the ‘Standard service’.

### Scenario analysis results

The scenario analysis results are presented in Table 5 below. The scenario analysis that resulted in the smallest effect on the ICER was increasing the EVLP rate to 20%. When the EVLP rate increased to 20%, costs and QALYs increased but the ICER was virtually unchanged as the increase in QALYs was negated by the proportionate increase in costs. Nevertheless, the number of transplants increased from 742 to 762 (2.6% increase).

**Table 5:** Scenario analysis results

Scenario number	Without EVLP				With EVLP					ICER	
	Cost	LYG	QALYs	Standard LTx	Cost	LYG	QALYs	Standard LTx	EVLP LTx	Cost per LYG	Cost per QALY gained
Base-case	£69,954	5.620	3.669	721	£73,964	5.660	3.714	675	67	£100,000	£90,000
1) Pre-trial transitions & 10%	£ 65,778	5.153	3.304	620	£69,265	5.199	3.354	586	59	£75,000	£71,000
2) EVLP rate 20%	£69,990	5.613	3.663	721	£77,128	5.691	3.747	635	127	£91,000	£85,000
3) Conversion rate 45%	£70,014	5.631	3.679	721	£73,014	5.673	3.725	675	67	£71,000	£65,000
4) 1:1 conversion rate	£70,136	5.634	3.675	721	£71,300	5.675	3.720	675	67	£28,000	£26,000
5) Utilities from literature	£69,851	5.606	3.937	721	£73,941	5.647	4.020	675	67	£98,000	£49,000
6) Increase in standard LTx in trial	£65,439	5.010	3.200	620	£73,377	5.500	3.590	721	0	£16,000	£20,000
7) DEVELOP-UK one-year survival post-transplant	£72,050	5.630	3.670	721	£76,128	5.620	3.680	675	67	Standard dominant	£408,000
8) Same post-transplant costs plus EVLP process costs in EVLP arm	£69,970	5.631	3.673	721	£71,569	5.674	3.720	675	67	£37,000	£34,000
9) Increased waiting list costs (120%)	£82,531	5.628	3.674	721	£83,742	5.671	3.721	675	67	£28,124	£25,855

*EVLP Ex-vivo lung perfusion, *ICER* Incremental cost-effectiveness ratio, *LYG* Life-years gained, *LTx* Lung transplant, *QALYs* Quality-adjusted life-years

**Fig. 4:**
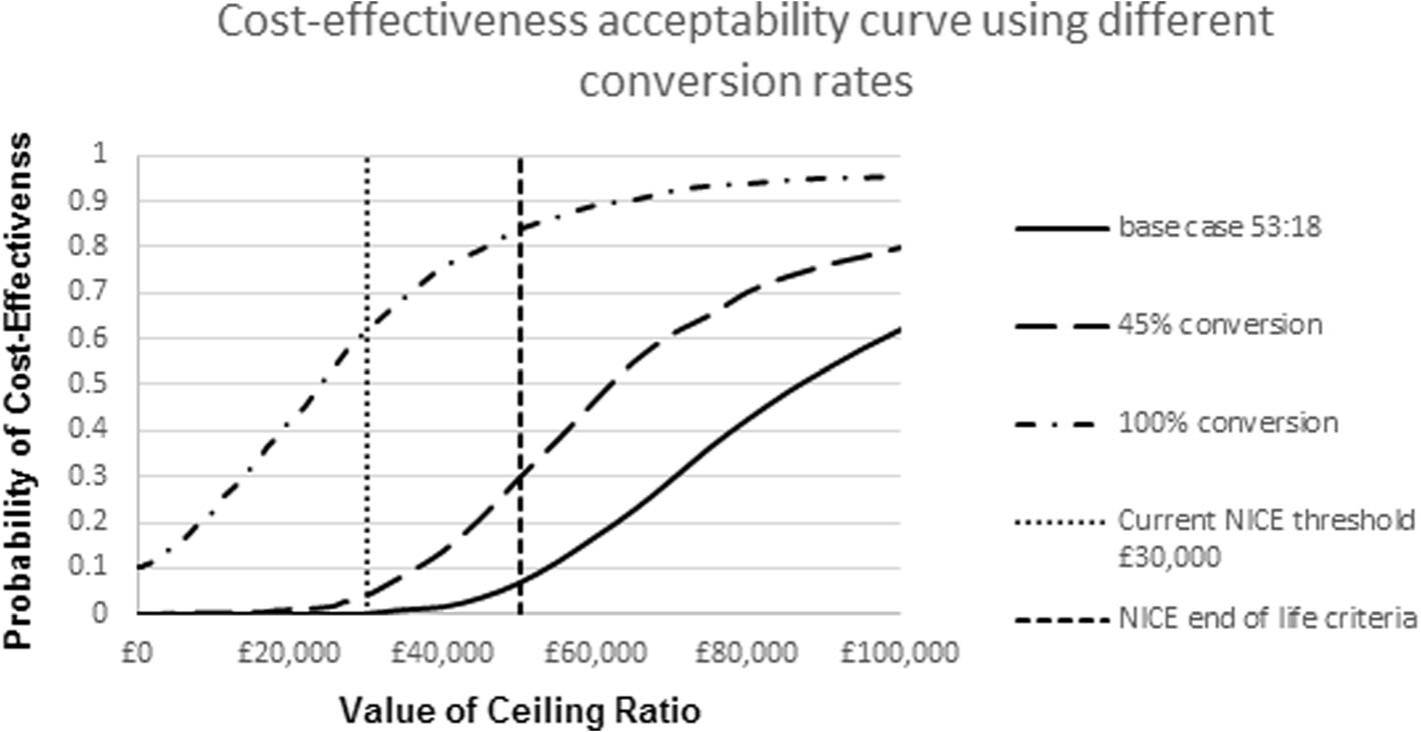
Cost-effectiveness acceptability curve: varying conversion rates

Changing the conversion rate from base-case (53:18 or 35%) to 45% decreased the ICER to £65,000/QALY gained. However, changing the conversion rate for EVLP to 1:1 from the base-case of 53:18 resulted in an ICER of £26,000/QALY which is at the upper end of what society might be willing to pay for a QALY in the UK [8]. These results are illustrated using a cost-effectiveness acceptability curve in Fig. 4 below.

In Scenario 8, when we used the same costs for post-transplant care (as reported in other EVLP settings), the ICER decreases to £34,000. Increasing the waiting list cost by 120% resulted in an ICER of £26,000/QALY. The scenario with the lowest ICER was Scenario 6; evaluating the unexpected increase in LTx during the trial, and this resulted in an ICER of £20,000/QALY. The scenario with the greatest effect on the ICER was scenario 7, resulting in an ICER of £408,000/QALY.

### Exploratory analysis

As pointed out in the *Results* section 3.1, the number of standard LTxs will differ between the two services after the first cycle as a result of the increase in transplants due to EVLP transplants. An exploratory analysis was carried out to estimate the effect on the ICER of relaxing this conservative assumption. If adopting a service including EVLP increased the number of EVLP lungs and at least maintained the number of standard lungs transplanted then the net cost per patient compared to not being transplanted would be £56,200 and the mean gain in QALYs would be 4.09. Assuming there would be an extra 46 standard transplants (based on the above exploratory analysis), then the incremental cost of service including both standard and EVLP transplants, compared with a service comprising just standard LTxs, would be £29,000 per patient, considered cost-effective.

Our second exploratory analysis relaxes the base-case assumption that costs and QALYs are not accrued to patients removed from the waiting list. The results and parameters from this exploratory analysis are presented in Table 6 below. Six more patients were removed from the waiting list in the ‘Standard service’ compared to ‘EVLP service’. Including the costs and QALYs of these patients does not affect the results; the ICER remains at £90,000/QALY.

**Table 6:** Results of including patients who are removed from the waiting list

Results of exploratory analysis							
	Without EVLP		With EVLP				
Survival after removal from waiting list	Cost	QALYs	Cost	QALYs	ICER	Cost source	QALY source
One month	£69,955	3.67	£73,964	3.71	£90,000	Dzingina [28]	DEVELOP-UK [4]
	£69,955	3.67	£73,964	3.71	£90,000	Dzingina [28]	Anyanwu [14]
	£69,956	3.67	£73,964	3.71	£90,000	Georghiou [29]	DEVELOP-UK [4]
	£69,956	3.67	£73,964	3.71	£90,000	Georghiou [29]	Anyanwu [14]
Three months	£69,958	3.67	£73,964	3.71	£90,000	Dzingina [28]	DEVELOP-UK [4]
	£69,958	3.67	£73,964	3.71	£90,000	Dzingina [28]	Anyanwu [14]
	£69,959	3.67	£73,964	3.71	£90,000	Georghiou [29]	DEVELOP-UK [4]
	£69,959	3.67	£73,964	3.71	£90,000	Georghiou [29]	Anyanwu [14]
Six months	£69,961	3.67	£73,964	3.71	£90,000	Dzingina [28]	DEVELOP-UK [4]
	£69,961	3.67	£73,964	3.71	£90,000	Dzingina [28]	Anyanwu [14]
	£69,964	3.67	£73,964	3.71	£90,000	Georghiou [29]	DEVELOP-UK [4]
	£69,964	3.67	£73,964	3.71	£90,000	Georghiou [29]	Anyanwu [14]
Parameters							
Source	Disease area		Parameter type			Parameter value	
Dzingina 2017	Advanced chronic disease		Palliative care cost per month			£1173	
Georghiou 2014	Any palliative care		Palliative care cost per month			£1644	
DEVELOP-UK	Lung transplant		Quality adjusted life-years			0.563	
Anyanwu	Lung transplant		Quality adjusted life-years			0.31	

*EVLP Ex-vivo lung perfusion, *ICER* Incremental cost-effectiveness ratio, *QALYs* Quality-adjusted life-years

## Discussion

### Main findings

According to the findings of the study, the estimated ICER for the EVLP procedure was £90,000/QALY suggesting that at the current NHS cost-effectiveness threshold, the ‘EVLP service’ would not be considered cost-effective [9], although it might be approved based on the NICE end-of-life criteria [8]. In the scenario analyses, the estimated ICERs were relatively stable for pre-trial transitions and EVLP rate adjustment. Changing the conversion rate (of substandard lungs to transplantable lungs) to 45% and using utilities identified from the literature increased the change in the ICER. The greatest variation on ICERs was using DEVELOP-UK one-year survival post-transplant, however, it should be noted that no evidence of a difference in survival between EVLP and standard LTx has been reported in previous trials. Four scenario analyses suggested that an EVLP service might be considered cost-effective, the ICER was reduced to below £30,000 for three of these: when applying only the increase in standard LTx witnessed during the trial, assuming a 1:1 conversion rate and increasing waiting list costs by 120%. Applying similar costs post-transplant forboth transplant types, as witnessed in other EVLP settings, resulted in an ICER of £34,000 suggesting that an EVLP service might be considered cost-effective.

The increase in standard transplants during the DEVELOP-UK study suggests that having access to the EVLP procedure increased recovery rates for what would previously have been deemed substandard lungs but were subsequently found to be suitable for standard transplant. The lesson is that many lungs that might initially be deemed unusable on referral may be deemed suitable on closer inspection using standard methods alone and without need for EVLP. However, this should be viewed with caution as the increase in standard LTx rates during the trial may have also resulted from other non-defined factors. EVLP assessments that did not result in a transplant were included in the study costs, however, this method was not adopted for the standard lung recovery as this information was not available. Including the costs of retrieving lungs that were not used for standard LTx could potentially alter the ICER between the two transplant procedures. The conversion rate for EVLP witnessed during the DEVELOP-UK study (53:18) was lower than the anticipated (40–50%) and lower than the two previous largest EVLP trials, which had conversion rates of 86% [30] from 58 EVLP assessments and 82% from 125 assessments [31]. The low conversion rate is likely a result of issues with donor lung selection for EVLP, the inflexibility of the multi-centre protocol. Furthermore within the context of a multicentre clinical trial donor lung selection is predefined whereas data drawn from the experience of a single centre are likely to be more selective in the choice of donor to perform EVLP on.

### Strengths and limitations

This is the first known economic evaluation of the EVLP technique for lung transplantation based on a multi-centre study. The strengths of this study were that all parameters used in the model were based on the UK adult LTx population. In addition, International Society for Pharmacoeconomics and Outcomes Research guidelines [32] were followed when designing the model reflecting best practice, while uncertainty in the data was evaluated using both deterministic and probabilistic sensitivity analyses. Finally, the DEVELOP-UK population is the general UK population as the study included a major proportion (> 70%) of all the lung transplantation activity over a two-year period. Therefore, the conclusions are totally relevant to UK practice.

The main limitation of the study was the low number of EVLP transplants during DEVELOP-UK meaning the outcome and use of services consequent to EVLP was imprecise and potentially unreliable. However, in the absence of other EVLP LTx data, and with DEVELOP-UK data subjected to PSA, deterministic analysis and scenario analysis, this study presents useful evidence on the potential for the EVLP technique. Evidence of higher resource use post-transplant by EVLP lung recipients in DEVELOP-UK has not been witnessed elsewhere, however this was explored in the scenario analyses by using similar costs post-transplant for both types of transplant. One model assumption was that when a patient in the ‘Waiting list’ state transitioned to the ‘Removed from waiting list’ state, no further costs or benefits were incurred. This assumption is the same as assumptions made in a Dutch economic evaluation of LTx service [33]. The cause of a removal is not known but it is reasonable to presume that most patients will be removed due to declining health. This, however, would result in higher costs and lower utilities until death. As a result, the costs are potentially underestimated and utilities overestimated in both arms; the net impact on cost-effectiveness is not possible to be predicted without information to model the costs and utilities of patients who are removed from the waiting list, however, this was mitigated by conducting the exploratory analysis which found including costs and utilities in the removed from waiting list state did not alter the ICER.

The use of published utility rates resulted in the third largest impact on the ICER of the sensitivity analyses reported. However, caution should be exercised as the published utilities came from a 1998 cohort, since then care for patients, as well as the criteria for registering on the LTx waiting list may have changed. Waiting list costs used in the base-case analysis were taken from the same cohort and are unlikely to reflect the increased use of high cost therapies for CF and IPF available when DEVELOP-UK was conducted. The authors were unable to locate more recent UK waiting list costs for use in the model. Around 50% of the DEVELOP-UK cohort had either CF or IPF, however, so waiting list costs during DEVELOP-UK were likely higher, a scenario analysis was conducted to explore the effect on the ICER of increased waiting list costs.

It should be noted that this evaluation is based on the UK LTx service and associated costs, therefore, whilst the model could be applied in other jurisdictions, the analysis may have limited applicability to health systems with a markedly different cost structure to the UK. The underpinning resource use data (available from the authors) may be more transferable.

### Comparison to other clinical evidence and future research

Despite a literature search, the authors are unaware of any other cost-effectiveness studies of EVLP LTx compared to standard LTx. One UK-based study was identified,from 1999, comparing standard LTx to medical treatment, This study reported total costs of £110,078 [14]. This is higher than the £69,954 mean standard LTx cost in our study, most of this difference results from higher post-transplant costs in the 1999 paper. This difference is likely due to several factors; early post-transplant results are better now than 20 years ago, as is overall survival and LTx centres are, when the patients are stable, also more comfortable with less frequent (and hence less costly) follow-up compared to 20 years ago.

This study has highlighted the need for more research in this area. Of particular interest are post-operative care resources, utilities and survival of EVLP recipients.

## Conclusion

The aim of the economic evaluation of the DEVELOP-UK study was to determine the cost-effectiveness of a UK adult LTx service including EVLP compared to one without, and the objective of including EVLP was to increase the pool of donor lungs. The results of this study suggest that combining EVLP with standard transplants increases the number of transplants, thereby reducing numbers on the waiting list. However, with the increased cost of transplant due to the EVLP procedure and the increased post-operative care costs the EVLP service is unlikely to currently be considered cost-effective, unless EVLP is deemed within the NICE remit for end-of-life care. Nevertheless, with an improved conversion rate, nearer to those witnessed in previous trials [30, 31], with a lower complication rate post-transplant for EVLP transplants, witnessed in other EVLP settings, and with higher waiting list costs, including EVLP in the UK LTx service the picture could be very different.

## Abbreviations

BMI: Body mass index
BNF: British National Formulary
CEAC: Cost-effectiveness acceptability curve
EVLP: Ex-Vivo Lung Perfusion
FEV1: Forced expiratory volume in 1 s
HTA: Health Technology Assessment
ICER: Incremental cost-effectiveness ratio
ISD: Information Services Division Scotland
LTx: Lung transplant
NHSBT: NHS Blood and Transplant
NICE: The National institute for Health and Care Excellence DEVELOP-UK A Study of Donor Ex-Vivo Lung Perfusion in UK Lung Transplantation
NIHR: The National Institute for Health Research
NK: Not known
PSSRU: Personal Social Services Research Unit
QALY: Quality adjusted life-year
